# Heterogenous wealth effects of minimum unit price on purchase of alcohol: Evidence using scanner data

**DOI:** 10.1371/journal.pone.0225538

**Published:** 2019-12-05

**Authors:** Anurag Sharma, Brian Vandenberg

**Affiliations:** 1 School of Public Health and Community Medicine, University of New South Wales, Sydney, Australia; 2 School of Social Sciences, Monash University, Australia; University of Miami, UNITED STATES

## Abstract

One of the key arguments given to oppose the “sin taxes” is that they are regressive in nature and place disproportionately higher cost on the poor thereby reducing their net wealth. The response to a reduction in net wealth attributed to tax can potentially have significant effects through an increase in alcohol purchase by heavy drinkers reinforcing or even offsetting the direct price or substitution effect of these taxes in reducing alcohol consumption. Comparatively little is known empirically about the net wealth effect associated with changes in alcohol tax policy, and this study aims to help fill this gap in the literature. In this study we aim to estimate how the wealth effects of introducing a minimum unit price (MUP) of A$2.00 per standard drink vary over the distribution (quantiles) of alcohol consumers. The data used in this study is a longitudinal panel of 1,395 households’ daily alcohol purchases (scanner data) recorded over a full year. Our analysis involves (i) quantile regression to estimate income elasticity over the distribution of consumption, and (ii) using these elasticities to estimate the potential wealth effects of a hypothetical change in alcohol prices from introducing an MUP policy. We control for consumer demographic characteristics, alcohol product prices and prices of close substitutes, and quarterly seasonal effects. We find that the estimated wealth effect from increasing the price of alcohol under a MUP policy is not significant at any point over the distribution of alcohol consumers. The policy increases per capita tax impact by less than A$5.00 per week for light/moderate consumers (50th—80th quantile) and decreases their daily per capita alcohol consumption by less than 0.02 standard drinks. Wealth effects attributable to an MUP policy are likely to be negligible. Substitution effects of the policy dominate the wealth effects in generating key health related outcomes such as reductions in alcohol consumption.

## 1 Introduction

A large body of scientific research in economics and public health consistently shows that alcohol taxation and pricing policies are among the most effective approaches for reducing overall consumption, heavy drinking, and alcohol related harm [1, 2]. These policies are particularly effective when they lead to an increase in the cost of the cheapest alcohol, which is commonly favoured by the heaviest consumers [3–8]. While conventional wisdom might suggest that alcohol consumption is habit forming and addictive, and thus resistant to price increases, the empirical evidence overwhelmingly shows that price rises do reduce consumption, even among heavy drinkers [9]. Today, governments in virtually all OECD countries impose some form of tax and/or price control on alcoholic beverages, and these are increasingly seen as important measures to improve public health (OECD, 2015). Accordingly, implementation of health promoting taxation and pricing policies is encouraged by the World Health Organisation (WHO) as part of comprehensive country-level strategies to reduce the harmful use of alcohol [10].

An increase in the relative price of alcohol, leading to a substitution effect is a key mechanism of alcohol taxation and pricing policies in curbing overall demand, deterring uptake and reducing harmful use of alcohol, and continues to receive most of the attention in empirical studies. Systematic reviews and meta-analyses of substitution effects on alcohol consumption behaviour show that although alcohol is relatively price inelastic, a one per cent price increase will, on average, reduce demand for alcohol by around half of one per cent, ceteris paribus [9, 11]. However, as we show in this study, substitution effects represent only one component of the effect of taxation and pricing policies on the demand for alcohol. The other component, is transmitted through the effect of increases in price on net wealth or consumer’s income and hence their purchasing power for alcohol—we call this the wealth effect. From a public health policy perspective, estimating the wealth effect attributable to the tax burden from alcohol taxes and other pricing policies is important because it has the potential to offset the reductions in alcohol consumption that are attributable to the substitution effect.

While there is general consensus in the literature that higher taxes and pricing policies reduce overall alcohol consumption in the population, attention is increasingly turning to the question of how such policies affect sub populations. Some recent evidence indicates that the substitution effects of alcohol taxation and pricing policies may place a disproportionate financial burden on light/moderate drinkers and low-income drinkers [12–14]. Opponents of such tax therefore claim that these policies are regressive, deterring the policymakers to implement comprehensive alcohol taxation reform. For example, recently the English government dropped the proposal for Minimum Unit Pricing due to concerns that it might penalise responsible drinkers and place a financial burden on poor without achieving its objective of reducing harmful drinking. The main reason cited by the government was that there is little empirical evidence on this issue. In particular, little is known empirically about how the wealth effect of alcohol taxation and pricing policies varies over the distribution of alcohol purchases and extent of regressivity of such taxes. This study aims to help fill this gap in the literature and make a timely contribution to inform policy making in the area. Our study estimates how the wealth effects of a $A2 MUP policy vary over quantiles of alcohol purchasing. We are able to examine whether the size of the tax burden relative to income is large enough to be considered regressive, and whether the financial impacts for light/moderate drinkers are unfair.

We analyse longitudinal scanner data comprising households’ socio-economic characteristics and daily records of their alcohol purchases over a full year, estimate the wealth effect due to tax burden on demand for alcohol, and apply this effect to a policy simulation. Using standard linear regression analysis we can estimate the mean wealth effect of alcohol taxation and pricing policies over a total sample distribution. However, this average effect provides only part of the picture, and might even be misleading, given the uneven distribution of alcohol purchasing and consumption. For example, a large proportion of households may consume only a small volume alcohol, and the average effect may unduly reflect their reactions. Previous studies using quantile regression show that the effect of alcohol prices varies considerably over the distribution of alcohol consumption [8, 15–17]. From a public health policy perspective, of most interest are the wealth effects of taxes and subsequent behaviour changes among those who purchase the largest volume of alcohol, and hence are at the greatest risk of harm. Thus, it is critical for policy makers to know if, and to what extent, different consumers over the distribution are responsive to the wealth effects of tax and pricing policy changes.

Our empirical analysis consists of two parts: For the first part, we employ censored quantile regression (CQR) analysis, using the technique detailed by [18], to estimate the ex-ante relationship between alcohol purchases and household income over the conditional distribution of per capita alcohol purchases. Our detailed dataset allows us to control for shoppers’ demographic characteristics, alcohol prices and prices of close substitutes, and quarterly seasonal effects. This CQR technique for different levels (i.e. quantiles) of consumption is well suited to analysis where the sample distribution is heterogeneous (e.g. in our sample alcohol purchases are highly skewed to the right) and where the sample is censored at zero for a large number of observations (e.g. around two- fifths of households in our sample recorded zero purchases in some quarters). For the second part, which incorporates the CQR estimation, we use the counterfactual analysis techniques proposed by [19] to estimate the unconditional quantile treatment effects (UQTE) of a counterfactual distribution resulting from a simulated change to alcohol pricing policy in Australia.

The counterfactual policy we examine is the introduction of a A$2.00 per standard drink minimum unit price (MUP) on alcohol based on the 1.84 CAD MUP in the Saskatchewan province of Canada. Variants of MUP policies were initially implemented in Canada, and have been recently the subject of considerable scrutiny and debate in Europe and Australia. Theoretically it has been established that imposing MUP enables lower optimal tax rate that is more sensitive to elasticity of demand [20]. Australian government has considered MUP as a potential national strategy for reducing risky drinking in the population [21] and recently MUP of $1.30 was introduced in the Northern Territory. The Scottish Government passed legislation to implement a MUP policy four years ago, but a legal challenge delayed its implementation until May 2018 when a MUP of 50 pence per standard drink was introduced. Hence, our study has worldwide policy relevance. Our key outcome variables for the counterfactual analysis include: the estimated change in the alcohol tax burden per capita (A$ per quarter); and, the subsequent estimated change in alcohol purchase per capita (number of standard drinks (12.67 ml pure alcohol) purchased per quarter). Respectively, these outcomes illustrate the extent to which the estimated wealth effect of a simulated A$2.00 MUP policy will impose an additional tax burden over the unconditional distribution of alcohol purchasing, and the extent of any public health benefits in terms of reductions in alcohol consumption (in addition to those resulting from substitution effects) stemming from the wealth effect of the policy change.

This paper is organised as follows. In Section 2, we further outline the motivation for our study, including a description of the public health burden from alcohol under the current taxation regime, and provide an overview of the empirical literature of relevance to our study. In Section 3 we present the data and provide some descriptive statistics. The econometric methods are discussed in Section 4 and Section 5 presents the main results. Section 6 concludes.

## 2 Background

### 2.1 Alcohol consumption in Australia

Almost one third (31%) of the total burden of disease in Australia is preventable by modifying exposure to well-known risk factors (AIHW, 2016a). Among these factors, alcohol use is responsible for a large and increasing share of the total disease burden (5.1%), rising from 4th place in 2003 to 3rd place in 2011, following tobacco use (9.0%) and high body mass (5.5%). Per capita alcohol consumption levels in Australia have been relatively unchanged since the 1990s, estimated to be around 10 litres per year [22]. However, per capita estimates can mask considerable heterogeneity in consumption behaviours across the population. For example, while a large proportion (21.8%) of the Australian population aged 14+ years do not consume alcohol, and many drink in moderation, drinking at risky levels is common. Around one in five (18.2%) Australians aged 14+ exceed the lifetime risk guidelines (i.e. on average drink more than 2 standard drinks per day) (AIHW, 2014). Significantly, 90.0% of these risky drinkers report drinking at home as their usual place of consumption (AIHW, 2016b). There is also increasing Australian and international evidence of “pre-loading” behaviour (drinking at home to achieve intoxication prior to drinking at licensed premises) that it is often motivated by the availability of relatively cheap off-premises alcohol [23]. This highlights the value of research using alcohol expenditure data that includes households’ off-premises alcohol purchases (i.e. taken away for consumption) as we have for this study.

The prevalence of heavy drinking in Australia is also reflected in the uneven distribution of alcohol consumption across the drinking population, with the top ten per cent of drinkers responsible for more than half (53.2%) of the total volume of alcohol consumed [24]. It follows that policies which can affect a downward shift in consumption among the heaviest drinkers, even if relatively small in percentage terms, will contribute to a substantial decrease in total population consumption, prevent exposure to risky levels of alcohol, and reduce much of burden of disease attributable to alcohol. This underlines the importance of estimating how alcohol policies impact not only on the average level of alcohol purchasing in the population, but also at each point (i.e. quantiles) over the conditional distribution of purchases.

### 2.2 Taxation and pricing policies in Australia

**Fig 1:**
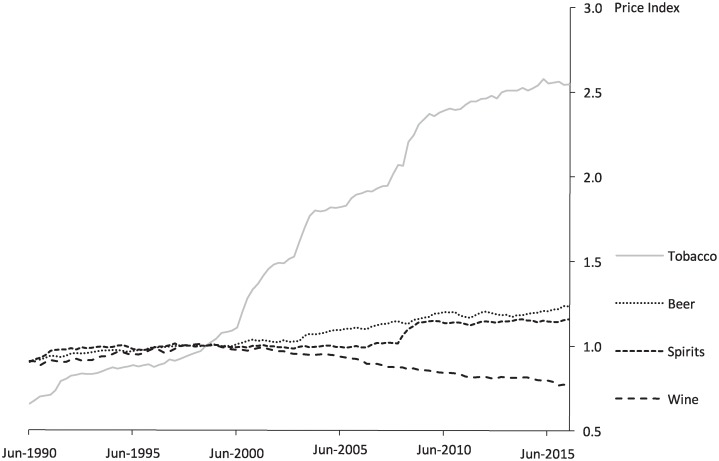
Real price of tobacco and alcohol products, Australia, 1990-2016.

In Australia, taxation of alcohol is the responsibility of the federal government which has two regimens: (i) a value-based “ad valorem” tax for wine and traditional cider, and (ii) 16 different specific tax rates (excise duties on domestic products and equivalent customs duties on imported products) for beer, spirits, brandy, and other excisable alcohol. The different tax treatments across beverage categories contribute to inconsistencies in the tax component of alcohol prices, and reduce the effectiveness of the alcohol tax system as a public health measure. A brief comparison of the recent history in alcohol and tobacco taxation policy in Australia highlights other shortcomings of the former as an effective public health strategy. For instance, while base tax rates on alcohol have been almost unchanged since 2000, with the exception of an increase in the base rate for ‘alcopops’ (pre-mixed spirits) in April 2008, the base tax rate on tobacco products has been adjusted upwards several times since 2000. As shown in Fig 1, this is reflected in large real increases in the price of tobacco products over the past two-decades. In contrast, the real price of alcohol has only slightly increased in the case of beer and spirits, and in the case of wine products the real price has declined. This illustrates the inadequacies of the current alcohol taxation system in increasing real prices to deter risky drinking. These trends in the real price of alcohol can be seen reflected in Australian drinkers’ attitudes and behaviour towards alcohol consumption, with only 6.3% of lifetime risky drinkers in 2013 reporting that the “increased price of a usual drink” is a reason for reducing their alcohol consumption; a statistically significant decline from 9.8% in 2010 (AIHW, 2016b).

Recent government-commissioned and independent reviews of Australia’s alcohol taxation system, along with parliamentary inquiries into alcohol-related harm, have consistently recommended reforms to increase the effectiveness of the system in reducing the harmful use of alcohol (Preventative Health Taskforce, 2009; AFTS, 2010; House of Representatives, 2015). One of the most important recommendations, from a public health perspective, is that the Australian government explore the feasibility of introducing a minimum unit price (MUP) for alcohol (Preventative Health Taskforce, 2009). This would establish a government-regulated floor price for a specified volume of pure alcohol or alcoholic beverage below which products may not be sold. The main effect of a MUP policy is to limit the availability of very cheap alcohol. Evidence of the effects of a MUP on consumption and harms is largely limited to either: (i) the experiences of Canadian provinces where versions of a MUP have been implemented and evaluated [25–27], or (ii) modelling studies that estimate the effects of a MUP if it were introduced in the UK [13, 28–30] and Australia [6, 17, 31]. The empirical evidence from Canada of the beneficial public health impacts of MUP policies is perhaps the most compelling. For example, a study by [25] of the government regulated minimum alcohol prices in British Columbia found that a 10.0% increase in the average minimum price of all alcoholic beverages was associated with an 8.95% decrease in acute alcohol-attributable admissions and a 9.22% reduction in chronic alcohol-attributable admissions 2 years later. However, despite the predicted benefits to population health, concerns are raised that such policies may impose a tax burden on individuals and households that is disproportionate to their consumption level, particularly for light and moderate consumers [21]. There are also important questions about whether heavy drinkers will be responsive to the income effects generated by MUP policies, given the habit forming and addictive nature of alcohol. Our study provides evidence to contribute to this debate and inform policy discussions.

### 2.3 Previous research

The literature most relevant to our study relates to the existing evidence on how alcohol taxation and pricing policies affect drinkers with different levels of consumption and income. For example, studies from the USA [12] and UK [13] show that the heaviest consumers, irrespective of household income, usually incur the highest alcohol tax burden in both nominal value and as a proportion of household income. Conversely, those who consume alcohol at low and moderate levels generally incur a relatively small tax burden. While these studies do not estimate wealth effects associated with tax burden, they highlight the strong association between a consumer’s level of alcohol consumption and the likely magnitude of wealth effects from taxation and pricing policies. This brings us to the main question of our study, which is how wealth effects vary by levels of alcohol purchasing. Only a very small number of studies have estimated consumer responsiveness over the distribution of purchasing (or consumption), and these generally focus on the substitution effect only [8]. One exception is the seminal study by [16], who use quantile regression techniques to estimate both price and income elasticities over the consumption distribution. They use US survey data that includes individuals’ drinking behaviour (with two-fifths of the sample reporting zero consumption), and socio-economics characteristics, which they probability match with a separate dataset containing alcohol price information. They report a small but significant income elasticity for being a drinker of 0.19, and find that income elasticities of demand for alcohol generally increase in size from 0.19 for the 5th quantile (of drinkers only) up to 0.30 for the 80th quantile, before then decreasing at the 90th quantile (0.26) and 95th quantile (0.24) (i.e. an inverted U-shape when plotted). In other words, they find that the heaviest drinkers are slightly less responsive than most to income effects. With regards to the estimated price elasticity of demand, they find that the heaviest drinkers (e.g. 90th quantile) are significantly less responsive than moderate drinkers (e.g. 50th quantile), with price elasticities of -0.49 and -1.19, respectively.

[15] use Australian data and a similar quantile regression technique to [16] to attempt replicating these results, but in contrast find large price elasticities overall (-0.96), with the largest price elasticities among drinkers near the upper end of the distribution: -1.26 for the 90th quantile (Note: they do not report income elasticities). Similarly, another recent Australian study by [17], also using quantile regression with the same data we use in this study, estimate substitution effects from a price change (ceteris paribus) and find that behavioural responses to a price increase from a simulated MUP policy is greater among consumers in the 90th to 97th quantiles than among those close to the median. These contrasting findings possibly reflect differences in data sources, time periods, and locations. This highlights the importance of using multiple sources of contextually relevant evidence to inform policy rather than generalising widely from a single study that may be inapplicable to the given policy setting and drinking culture.

## 3 Data

### 3.1 Homescan data

A detailed description of the data used in this study is provided in several earlier studies (for example see [17] among others). We therefore provide only general details here, and if necessary some detailed information that is unique to this study. Households on the consumer panel are recruited via third party vendors such as websites, blogs, social media etc. and participation is voluntary. Households that register to participate have to complete a detailed questionnaire on household demographics. This information is then used to select the households on the panel via a stratified random sampling. Finally households are selected based on their household characteristics to balance the panel to be representative of the population. A number of reviews highlight limitations of data commonly used in this field of research, and recommend that models be built using datasets that include household and/or individual alcohol spending and consumption, product details, date and location of purchase and the price paid per product [10, 32, 33]. With regards to consumption data, a review by [32] recommended that transaction level detail of consumers’ alcohol purchasing is critical for accurate estimation of consumption and measurement of policy effects. Similarly, with regard to alcohol price data, a review by [33] reported that scanner data (i.e. prices recorded by retailers or consumers directly scanning the barcode of a product) are the most accurate and reliable data for analysing the effects of alcohol price changes. While access to this type of data is usually rare, for this study we use a sample of consumer scanner data for Australian households’ daily alcohol purchases over a full year, obtained from the Nielsen Company’s continuous HomeScan panel survey. The data are collected from a sample of demographically representative households over a period of 1 year (between January 2010 and January 2011) residing within Victoria, the second most populous State of Australia. Each household is provided with a scanning device and required to scan and record each item purchased and taken home from different retail locations (supermarkets, grocery stores, local shops, etc.) over the specified time period. Hence, the data include a high level of specificity on individual household alcohol purchases not provided in publicly available population survey datasets, such as alcohol type, brand, flavour variant, size (litres of beverage and litres of alcohol), quantity, packaging (e.g. multi-pack), price paid per item (A$), total spend per shopping trip, and the date and location (i.e. store name) of the shopping trip, along with demographic and economic information about the individual household and the shopper. Using individual product label information obtained from our own manual store survey we impute the alcohol content (% alcohol by volume) for each product to calculate the litres of alcohol and the number of standard drinks.

HomeScan data has been used extensively for research into consumer behavior in relation to food and non-alcoholic beverages [34–36] but has only been used in a small number of alcohol studies [14, 17, 37, 38]. A panel expenditure dataset such as HomeScan has considerable appeal over other datasets used for studying alcohol purchasing behaviour. Alcohol researchers and policy makers usually rely upon periodic, self-report population surveys of household alcohol expenditure or drinking patterns to monitor and analyse alcohol consumption. The limitations of such surveys are well documented and include sampling bias, response bias, measurement bias, and recall bias, with under-reporting of consumption by heavy drinkers seen as a common weakness [10]. Annual estimates of national per capita consumption of alcohol based on sales or taxation data provide a more reliable indicator of total consumption, but in many jurisdictions these are reported at a national population level only, thus constraining their usefulness for studying consumer behaviour in detail. The appeal of HomeScan panel data, therefore, is that it overcomes many limitations of existing surveys by collecting information on each household’s alcohol purchasing constantly over 52-weeks, and includes disaggregated detail about daily shopping trips and individual products purchased by each household. A validation study of HomeScan data in the US found that households reported single purchases 99% accurately and multiple purchases 86% accurately (when checked against stores’ sales records), and the small level of recording errors is similar to other datasets for which cross-validation studies have been undertaken [39]. The design of HomeScan surveys is similar across countries, thus potentially enabling replication of our study elsewhere and some comparison of findings. The Australian panel is built using a sampling frame based on Australian Bureau of Statistics’ (ABS) information on the geographic, demographic, social and economic distribution of the Australian population. We estimate that, on average, our sample of HomeScan data accounts for around two-thirds (65 per cent) of households’ total alcohol purchases, given that: (i) off-premises alcohol represents an estimated 79 per cent of the total alcohol volume purchased in Australia [40], and; (ii) for the majority (81 per cent) of drinkers in Australia, their usual place of alcohol consumption is in their own home (AIHW, 2014).

### 3.2 Alcohol purchases

Using the detailed product level information in our data for each daily recorded alcohol purchase over 12 months, we calculate the total number of standard drinks (12.67mL of pure alcohol) purchased by each household in each quarter. We use this information to derive the average number of standard drinks purchased per adult household member per day, which is our key outcome variable of interest. Although this variable is not a measure of individual alcohol intake, it is associated with very precise information on product characteristics (see detail on calculation method in supporting information—S1 Appendix). In addition, there is variation in prices over time and space in our sample which we exploit for identification of demand. An advantage of using quarterly aggregations, as the meta-analysis by [41] shows, is that they are sufficiently long to wash out the effects of short-term inventory behaviour by consumers that can inflate elasticity estimates.

### 3.3 Control variables: Prices and shopper characteristics

One standard approach to construct household-specific quarterly unit prices for our study, following the literature [42], would be to simply take the ratio of household quarterly alcohol expenditure on the quarterly quantity of standard drinks purchased. However, as [43] discuss, time-invariant variables that are omitted from our estimation model and correlated with prices (e.g. quality preferences) can lead to endogeneity bias (confounding effect). Quality preferences for alcohol are likely to vary considerably between individuals, affecting both price paid and quantity of alcohol purchased. For example, some households may face a quality-quantity trade-off for each beverage and may choose for cheaper brands if they prefer quantity over quality. Similarly, higher unit values may correspond to products of better quality and, as such, are likely to reflect household preference for quality. Hence, we follow approach suggested by [43], originating with [44], to eliminate quality-related variations in alcohol prices by constructing a Laspeyres price index *p*_*ct*_ for households *h* living in postcode *c* at quarter *t* from the unit prices of products, where the latter is defined as a brand in an alcohol category:

$$\begin{array}{c} p_{ct}=\frac{\sum_{k}p_{kct}q_{k0}}{\sum_{k}p_{k0}q_{k0}} \end{array}$$ (1)

where *k* denotes products, *p*_*kct*_ is the unit price of *k* in postcode *c* at quarter *t* and and *p*_*k*0_ and *q*_*k*0_ are the sample median prices and quantities for *k*. This approach aims to ensure that the derived price index will not vary with systematic differences in unobserved household characteristics, which may either affect preferences for quality or influence local prices. In the demand function estimate, we also control for the prices of the closest substitutes, including: regular soft drinks; diet drinks; fruit juice; and, bottled water. We construct price indices for these other beverage categories as per the procedure we use for alcohol, and assume that these price indices remain constant in the counterfactual scenario.

### 3.4 Descriptive statistics

**Fig 2:**
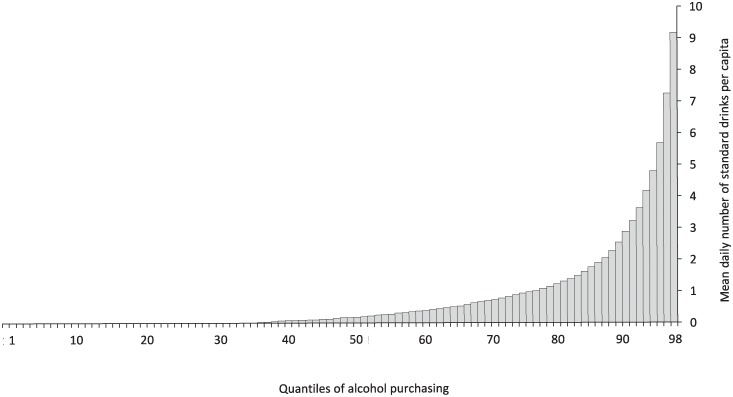
Unconditional quantile distribution of alcohol purchasing.

**Fig 3:**
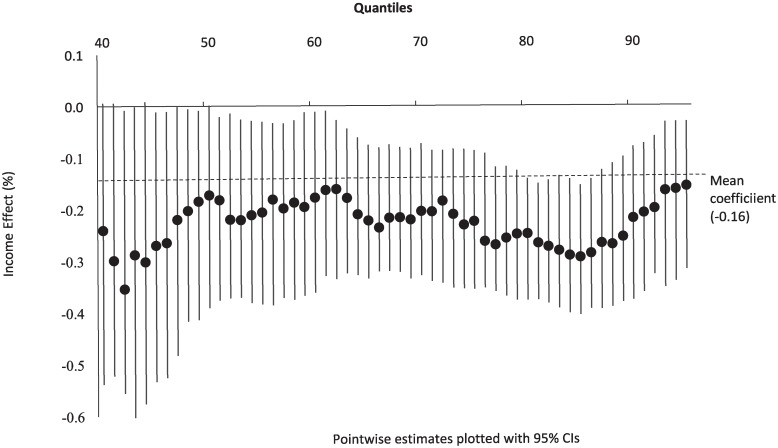
Income elasticities: Conditional quantile distribution of alcohol purchasing.

The HomeScan dataset includes various social and demographic characteristics of each household such as, for example, the gender of the household’s main shopper. This information is collected annually, and hence does not vary by quarters. We also include three quarter dummies to control for seasonal variations in demand. The balanced panel comprises of 1395 households—who participated in the panel for a full, continuous 52-week period, from 24 January 2010 to 22 January 2011. Of these, we retain only those households who made at least one alcohol purchase in at least one of four quarters (n = 878; total household-quarter observations = 3,512). Hence, a household’s alcohol purchasing volume is censored at zero in quarters where they did not purchase any alcohol. Table 1 reports some descriptive statistics for the variables in our sample, tabulated by decile of alcohol purchasing (based on per capita household volume per quarter). An uneven distribution of alcohol purchasing over the sample is clearly apparent (see Fig 2) and, as shown in the above table, is also mirrored by alcohol expenditure with the heaviest consumers (D10) spending more than double the amount per quarter on alcohol than light consumers (D5). However, on a A$ per standard drink basis, the heaviest consumers spend considerably less than light consumers (see Fig 3), and this aligns with the findings of other recent studies of off-premises alcohol purchasing behaviour in the Australian population [45].

**Table 1:** Summary statistics by distribution of alcohol purchases.

Deciles of alcohol purchases	D5	D6	D7	D8	D9	D10	Full Sample
**Alcohol purchasing**							
Per capita volume (std drinks/quarter)	8.39	21.11	41.82	76.83	144.63	561.6	85.43
Per capita expenditure (A$/quarter)	35.03	34.71	39.09	44.3	48.81	99.62	43.81
Price paid per standard drink (A$)	4.74	1.72	0.97	0.59	0.35	0.19	1.72
**Household characteristics**							
Income (A$/year)	68,364	65,618	67,769	67,424	63,538	57,103	65,135
Per capita income (A$/year)	28,419	26,393	27,247	29,239	23,304	22,839	26,747
Household size (persons)	2.7	2.77	2.72	2.62	2.72	2.48	2.7
Female head (% of households)	53.95	48.55	50.71	47.48	47.86	35.61	49.3
Young family (% of households)	18.64	20.52	20.8	17.66	17.38	14.25	18.91
Older family (% of households)	16.38	17.92	19.94	15.1	20.22	14.81	17.89
Non-metropolitan (% of households)	19.77	24.28	23.08	20.23	19.94	27.07	22.22
**Shopper characteristics**							
Age (years)	51.72	51.62	52.59	53.32	55.23	57.45	52.9
Female (% of shoppers)	79.66	78.9	77.77	73.79	76.63	58.4	76.08
Employed full-time (% of shoppers)	31.64	36.42	32.48	22.91	25.07	26.78	32.36
Employed part-time (% of shoppers)	32.49	30.92	30.2	26.5	32.19	22.51	30.53
Not working (% of shoppers)	35.88	32.66	37.32	43.59	42.74	50.71	37.11
Professional (% of shoppers)	25.42	23.7	20.8	20.23	14.53	10.54	20.96
**Seasonal effects**							
Quarter1 (% of total obs)	24.01	23.7	27.35	23.08	24.5	23.36	25
Quarter2 (% of total obs)	21.19	22.83	24.22	23.08	25.36	24.5	25
Quarter3 (% of total obs)	23.45	23.12	24.21	23.08	23.93	25.93	25
Observations	354	346	351	351	351	351	3511

Notes: D50 to D100 represent deciles of the unconditional distribution of alcohol purchases (number of standard drinks purchased per capita per quarter), ordered from low purchasing households (Q50) up to heaviest purchasing households (Q100). Std drinks (standard drinks) = 12.67 ml of alcohol (equal to 10gm of alcohol). A$ = Australian dollars. Values are averaged across all quarters, with the exception of seasonal effects.

Household income does not appear to vary systematically with volume of alcohol purchasing, though incomes for the heaviest consuming households (D10) are relatively lower than those in the lighter consuming deciles. On average, the heaviest consuming households also have relatively smaller household sizes, and a smaller proportion of these households include families (young or older). Three-quarters of shoppers in our sample are female, which is consistent with research in the UK showing that females are more likely than males to purchase off-premises alcohol [46]. Shoppers belonging to the lighter consuming households are more likely to be younger, female, employed full time, and employed in professional occupations, whereas shoppers belonging to heaviest consuming households are more likely to be older, male, and not working (i.e. unemployed, retired).

## 4 Methods

Our main aim is to capture heterogeneity i.e. how income changes/tax burden resulting from a MUP policy will affect the purchase of alcohol not just at sample mean but at all levels of alcohol consumption i.e. for light, moderate and heavy drinkers. This depends on how individuals change their alcohol purchase in response to changes in their income (also called income elasticity of each individual). As a first step we therefore use a quantile regression approach to estimate income elasticity of individuals at each quantile. Subsequently, we use these elasticities to estimate the simulated effect of a $2 MUP on alcohol purchasing. This approach uses the whole sample and assigns varying weight to each observation across quantiles to obtain quantile level estimates. One critical issue in our setting is that a large share of households did not purchase alcohol in some quarters and thus we have large number of zeros in the alcohol purchase data (also known as left-side censored data). Ignoring this censoring can lead to misleading estimates. We incorporate censoring in our empirical approach by estimating a censored quantile regression (CQR) model proposed by [18]. S1 Appendix provides detailed discussion of our methodology.

## 5 Results

As a first step, we model household’s per capita volume of alcohol purchasing as a function of per capita household income and controlling for characteristics of the household’s shopper, prices of alcohol and other non-alcoholic beverages, and seasonal factors. This provides us with the effect of income on alcohol consumption (ceteris paribus). Table 2 presents the results of our CQR model and Fig 3 (below) shows these graphically.

**Table 2:** Censored quantile regression: Income elasticities of log quantity of alcohol purchased.

	Q40	Q50	Q75	Q90	Q95
Log Income	-0.239	-0.172	-0.224*	-0.217*	-0.155*
	[-0.550 0.00]	[-0.394, 0.011]	[-0.367 -0.123]	[-0.377 -0.084]	[-0.292 -0.046]
**Log Prices**					
Alcohol	-0.385	-0.640*	-0.306*	-0.342*	-0.379*
	[-0.655 0.512]	[-0.812 -0.071]	[-0.434 -0.122]	[-0.826 -0.016]	[-0.883 -0.015]
SSBs	0.301	-0.004	-0.127	-0.188	-0.110
	[-0.566 1.153]	[-0.542, 0.631]	[-0.391 0.306]	[-0.320 0.296]	[-0.413 0.249]
Diet Drinks	0.540	0.889*	0.586*	0.386*	0.190*
	[0.00 1.63]	[0.486 1.424]	[0.255 1.039]	[0.160 0.698]	[-0.060 0.449]
Bottled water	-0.420	-0.729*	-0.268*	-0.292*	-0.152*
	[-0.956 0.218]	[-1.15 -0.250]	[-0.573 -0.080]	[-0.512 -0.054]	[-0.422 -0.051]
Fruit Juice	0.102	0.287	-0.122	-0.358	-0.613*
	[-0.661 1.078]	[-0.353 0.954]	[-0.245 0.660]	[-0.843 0.080]	[-0.960 -0.260]
Female Shopper	-1.25*	-0.881*	-0.690*	-0.869*	-0.804*
	[-1.72 -0.351]	[-1.231 -0.584]	[-0.871 -0.431]	[-1.055 -0.571]	[-0.950 -0.556]
Observations	3511	3511	3511	3511	3511

95% CIs in square brackets.

*: Significant effects at 5% significance level.

Results for additional covariates skipped for brevity.

On average, across the whole sample, the CQR model predicts that a one per cent increase in income leads to a -0.16 per cent change in alcohol consumption (ceteris paribus), and the estimate is significantly different from zero. However, importantly, the CQR results reveal considerable heterogeneity in income effects at different points over the conditional distribution of alcohol purchases. For example, most of the estimated income effects below median are not significantly different from zero, and hence, it appears that light consumers’ alcohol purchasing behaviour is not sensitive to income. However, of most relevance to policy from a public health perspective, are the effects we find for those who consume at the risky end of the distribution. For example, the estimated elasticity for 90th percentile shows that a one per cent increase in income among consumers (who purchase, on average, 2.43 standard drinks per day) would lead to a -0.22 per cent change in the volume of alcohol purchased. That is, the model predicts that a lower income is associated with relatively higher level of alcohol purchasing. Also noteworthy, is that above 72th percentile we find a U-shaped relationship over the distribution of consumption. That is, the size of the income response generally increases towards the upper end of the distribution and peaks at 85th percentile (-0.29 per cent), after which it decreases in size, back towards the overall mean estimate for the sample of -0.16 at 95th percentile. This relatively stronger, but somewhat varying response to income among many of the heaviest alcohol consumers would not have been detected using only standard mean regression, and thus illustrates the value of CQR analysis in evaluating policies aimed at reducing harmful use of alcohol. It should be noted that given the low magnitude of income elasticities, change in income one way or other will not lead to significant changes in alcohol purchase. For example even for households purchasing alcohol at risky levels (up to 45 standard drinks per week at 95th quantile) a 1% increase in income for example will reduce their purchase by just 0.16% (around 0.07 standard drinks per week).

### 5.1 Counterfactual analysis

The coefficients in our counterfactual analysis are derived from an unconditional quantile regression, which is a subtle, but important difference to the conditional quantile regression used in our CQR model to estimate the ex-ante wealth effects described above. The covariate effects from the unconditional quantile regression used in our counterfactual analysis represent the effect of changing the value of a key covariate of interest (i.e. per capita household income), but keeping the full distribution of all other covariates the same (e.g. gender of shopper, price indices). This is distinct from coefficients obtained by conditional quantile regression which represent the effect on the conditional quantile of the outcome distribution, conditioned on the mean values of other covariates [47].

Table A in S1 Appendix presents the unconditional quantile treatment effects (UQTE) from our counterfactual analysis: the introduction of a A$2.00 MUP policy. In summary, the UQTE at all quantiles are not significantly different from zero. That is, the results of the counterfactual analysis indicate that the MUP policy will not have significant wealth effects at any point over the unconditional distribution of alcohol purchases. This suggests that the perception among opponents of MUP policies that these measures will be regressive, by imposing a disproportionate burden on responsible drinkers (drinking at lower quantiles), can be rejected.

**Fig 4:**
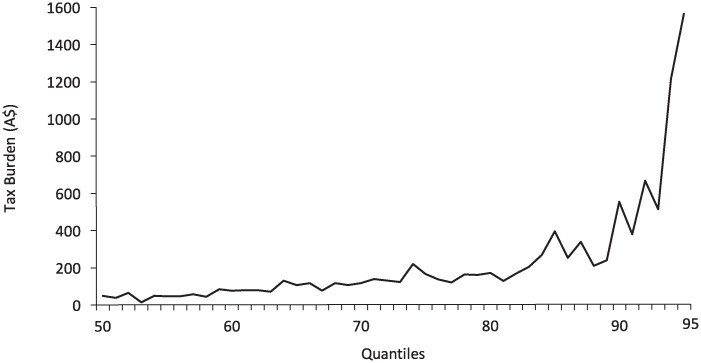
Additional annual tax burden per capita resulting from A$2 MUP policy.

We further explore the regressivity of MUP policy by using UQTE for each quantile to obtain the estimated additional annual per capita tax burden that consumers would face under a MUP policy. We use the term ‘tax burden’ to denote the additional alcohol purchase costs faced by consumers under a A$2.00 MUP policy, noting that although a MUP policy is not a tax, it is form of government price regulation that leads to a higher purchase costs for consumers, just as tax increases would lead to higher purchase costs. Please note this tax burden includes both price and income effects of MUP. The detailed derivation of tax burden is discussed in the S1 Appendix. The distribution of this additional tax burden is presented in Table B in S1 Appendix and is shown graphically in Fig 4 (below).

For all quantiles up to 85th percentile the additional tax burden from a MUP policy is small in nominal terms (¡A$1.00 per day) or less than 0.5% of income) indicating almost negligible financial impact of MUP policy on light, moderate, and the majority of heavy consumers. For heavier consumers (above 90th quantile) their tax burden under a MUP policy is predicted to be slightly higher than all other quantiles. This reflects the large volume of cheap alcohol that the heaviest consumers purchase (for example the mean price paid per standard drink by consumers in 90th quantile = A$0.18), the price of which would increase considerably under the MUP policy.

## 6 Discussion

The empirical analysis in this study uses an innovative method (censored quantile regressions) and unique scanner data to conclude that there are no significant wealth effects at any point over the distribution of alcohol purchases resulting from an increase in the price of cheap alcohol under a MUP policy. We show that despite concerns a MUP policy may have unfair impacts by disproportionately increasing the tax burden on light and moderate consumers compared to heavy consumers [21], the financial impacts would be negligible over the entire distribution. Similarly, the predicted change in alcohol purchases from the wealth effects of a MUP policy would be negligible over all points in distribution. Furthermore, the estimated wealth effects we find are considerably smaller in magnitude than the substitution effects estimated by [17] using the same data as ours, and by [13] in a UK modelling study. This suggests under a MUP policy it is the substitution effect rather than the wealth effect which is the dominant component of the total price effect in determining tax burden and influencing demand for alcohol. One of the key characteristics of alcohol as a commodity is that a reduction in income can sometimes lead to increases in its consumption especially among heavy and addictive drinkers. This has major implications for policies focussing on financial levers such as taxation of alcohol or MUP to reduce alcohol consumption. On the one hand an increase in price of alcohol can lead to reduction in alcohol purchases (standard price or substitution effect) but on the other hand tax burden of such policy results in reduction in income (so called wealth effect) and can potentially increase alcohol consumption thereby offsetting the effectiveness of taxation policies, a major concern among policy makers. Our findings help fill this evidence gap in the literature by suggesting that there is no evidence to support such concerns and MUP policy overall has potential of reducing alcohol consumption among risky drinkers. Methodologically, our approach highlights the value of counterfactual analysis techniques for thorough policy evaluation. That is, when examining policy effects on consumption behaviour that is unevenly distributed, such as alcohol consumption, it is critical to estimate the unconditional treatment effects at each point over the entire distribution, particularly where there is uncertainty about the differential effects between light, moderate and heavy consumers.

Some important caveats apply to our findings, in relation to limits of the data, the model, and the possible effects of factors we have not examined. As discussed earlier, our data includes records for households’ off-premises alcohol purchases only, and hence we are not able to compare the differences in the wealth effects for on- and off-premises alcohol over the distribution. Another obvious constraint of our household data is that it includes only limited individual-level information. For example, in order to derive a measure of purchases per capita, we assume that alcohol purchases are shared equally among adults in the households. This bias however will not affect our main findings as assigning say all purchase to a single adult will further increase the extent of substitution effect and reinforce our argument that contribution of wealth effects to change in alcohol consumption is almost insignificant relative to substitution effect. Another limitation is the possibility of inventory behaviour (i.e. stockpiling) in our sample, which we do not explicitly account for. However, meta- analyses [41] of the literature suggest that the effects of short-term inventory behaviour are likely to be washed-out using quarterly aggregations we have done in our analysis.

## Supporting information

S1 Appendix (PDF)
