# Supplementary material for: Heterogenous wealth effects of minimum unit price on purchase of alcohol: Evidence using scanner data
Source: PLoS One. 2019 Dec 5;14(12):e0225538. doi: 10.1371/journal.pone.0225538 (PMC6894865; doi:10.1371/journal.pone.0225538)
Supplement: S1 Appendix — (PDF) [file pone.0225538.s001.pdf]

S1 Appendix

A Generating Price Indices

We follow approach suggested by [1], originating with [2], to eliminate quality-related variations in alcohol prices by constructing a Laspeyres price index p_{ct} for households h living in postcode c at quarter t from the unit prices of products, where the latter is defined as a brand in an alcohol category:

$$p_{ct} = \frac{\sum_k p_{kct} q_{k0}}{\sum_k p_{k0} q_{k0}} \tag{1}$$

where k denotes products, p_{kct} is the unit price of k in postcode c at quarter t and and p_{k0} and q_{k0} are the sample median prices and quantities for k. This approach aims to ensure that the derived price index will not vary with systematic differences in unobserved household characteristics, which may either affect preferences for quality or influence local prices. In the demand function estimate, we also control for the prices of the closest substitutes, including: regular soft drinks; diet drinks; fruit juice; and, bottled water. We construct price indices for these other beverage categories as per the procedure we use for alcohol, and assume that these price indices remain constant in the counterfactual scenario.

B Quantile Regression

We use quantile regression to identify heterogeneity in ex-ante wealth effects across the distribution of alcohol purchasing. To begin, consider a standard linear regression model that defines the conditional mean of the dependent variable y, as a linear function of a vector of explanatory variables, x, written as

$$y_i = x_i^{'} \beta_\theta + \epsilon_i \;\; and \;\; E(y_i|x_i) = x_i^{'} \beta \tag{2}$$

where ϵ is an error term. While this standard linear regression is useful for estimating the average (mean) effect of x on y, it provides only a partial view of the relationship. Quantile regression provides a more complete picture by estimating the relationship between y and x at different points (i.e. quantiles) over the conditional distribution of y. That is, it allows for effects of the independent variables to differ over the quantiles (of alcohol purchases in our case), which is of particular interest to us given the uneven distribution of alcohol purchases in the sample population. The starting point for quantile regression is the conditional quantile function (CQF). The CQF at quantile θ for a continuously distributed variable y, given a vector of regressors x, can be defined as

$$Q_\theta(y_i|x_i) = x_i^{'} \beta_\theta \tag{3}$$

where $Q_\theta(y_i|x_i)$ denotes the θth conditional quantile of y_i. So, for example, to describe the median (mid-point in the distribution), we take $\theta = 0.5$. In the context of our study, where large volumes of alcohol are purchased by a relatively small share of households, the median is probably more informative than the mean. The quantile regression model of the form first introduced by [3] can written as:

$$y_i = x_i^{'} \beta_\theta + \epsilon_{\theta_i} \tag{4}$$

where β_θ is the vector of parameters, and the conditional quantile of the error term is zero. The quantile regression estimator of β_θ is found by solving the problem:

$$\min_{\beta_\theta} \frac{1}{N} \left\{ \sum_{y_i \geq x_i^{'}\beta_\theta} \theta|y_i - x_i^{'}\beta_\theta| + \sum_{y_i \geq x_i^{'}\beta_\theta} (1-\theta)|y_i - x_i^{'}\beta_\theta| \right\} = \min_{\beta_\theta} \frac{1}{N} \sum_{i=1} \rho_\theta(\epsilon_{\theta_i}) \quad (5)$$

where $\rho_\theta(\lambda) = (\theta - I(\lambda < 0))\lambda$ is the check function, and $I(\cdot)$ is the usual indicator function. The special case where $\theta = 0.5$ is called the median regression estimator, or the least absolute deviations (LAD) estimator. The minimisation problem in Eq. (5) can be solved by linear programming for different quantiles of the dependent variable (see [3]), which makes estimation relatively fast [4]. Additionally, the quantile regression estimator has several important equivariance properties that are preserved under monotone transformations, which help facilitate the computation procedure. For example, if we transform a set of positive observations by taking the values in logs, the median of the log will be the median of the untransformed data. In our sample there is left-side zero censoring because a large share of households did not purchase alcohol in some quarters. Therefore, quantile regression of the form described in Eq. (3) is not applicable and this specification needs to be corrected for zero censoring. One alternative is the tobit model, also referred to as the censored regression model, which can be written as:

$$y_i^* = x_i^{'}\beta + \epsilon_i \quad (6)$$

where

$$y_i = y_i^* \quad if \quad y_i^* > 0$$
$$y_i = 0 \quad if \quad y_i^* \leq 0$$

In other words, the tobit model is a standard regression model where all values of the dependent variable that are equal to, or less then, zero take the value zero. The tobit model describes both the probability that $y_i|x_i = 0$; and the distribution of $y_i|y_i > 0$. However, despite its popularity, [5] shows that if the errors are not normally distributed and homoscedastic, then the estimated coefficients of the tobit model are inconsistent.[1] [6] proposes an alternative to maximum likelihood estimation of the parameters of the tobit censored regression model that is not based upon strict parametric assumptions. His proposed censored least absolute deviations (LAD) estimator $\hat{\beta_n}$ is a generalization of LAD estimation for the standard linear model, and, unlike estimation methods based on the assumption of normally distributed error terms, the estimator is consistent and asymptotically normal for a wide class of error distributions, and is also robust to heteroscedasticity. The value of the estimator $\hat{\beta_n}$ can be found by solving:

$$\min_{\beta_\theta} \frac{1}{N} \left\{ \sum_{i=1}^{N} |y - max(0, x_i^{'}\beta_0)| \right\} \quad (7)$$

[6] later extended this median (LAD) regression, recognising that in situations where the dependent variable is heavily censored (i.e. where $y = 0$ for a large share of the observations), the censored LAD estimator $\hat{\beta_n}$ may be very imprecise, since the median of y_i would be uninformative about β_0 for much of the sample. In such a situation, Powell suggests it may be preferable to centre the distribution of y_i at a higher quantile

[1] When estimated coefficients are inconsistent it means that as the sample size increases indefinitely, the estimators do not converge to their true (population) values and, therefore, the reported standard errors and confidence intervals will be misleading.

than the median, because a higher quantile would be more often positive, and thus more often informative about β_0. [6] further shows that under certain regularity conditions, the estimators generated from a censored quantile regression (CQR) model are consistent, independent of the distribution of the error term, are asymptotically normally distributed, and are robust to outliers of the dependent variable. Below we describe the CQR model in detail.

B.1 Censored Quantile Regression

When the conditional quantile of the error term is zero, a CQR model of alcohol purchases censored at zero can be expressed as:

$$Q_\theta(y_i|x_i) = max\{0, Q_\theta(x_i^{'}\beta_\theta + \epsilon_i|x_i)\} = max\{0, x_i^{'}\beta_\theta\} \tag{8}$$

The CQR estimator of β_θ proposed by [6] is found by solving

$$\min_{\beta_\theta} \frac{1}{N} \left\{ \sum_{i=1}^{N} [\{\theta - I(y_i < max\{0, x_i^{'}\beta_\theta\})\}(y_i - max\{0, x_i^{'}\beta_\theta \right\})] \tag{9}$$

where I is an indicator function taking the value of unity when the expression holds, and zero otherwise. For observations when $x_i^{'}\beta_\theta$ is equal to or less than zero (i.e. zero being the censoring point), $max\{0, x_i^{'}\beta_\theta\} = 0$ and Eq. (7) is minimised by using only the observations for which $x_i^{'}\beta_\theta$ is greater than zero.

While Eq. (4) is a linear function and can be solved by linear programming as noted above, the expression $max\{0, x_i^{'}\beta_\theta\}$ in Eq. (8) is not linear and has no linear programming representation. Therefore, to solve Eq. (8), we use the three-step algorithm proposed by [7] for known censoring points that is simple, easily computable (comparable to linear least squares), well-behaved, robust, and performs well near the censoring point. [7]'s estimator $\hat{\beta}_\theta$ is obtained in the following three steps. First, the censoring probabilities are estimated by a parametric classification (probability) model $\delta_i = pX_i^{'}\gamma + \epsilon_i$, where δ_i is the indicator of no censoring. Then, for each quantile regression, a sample of observations with sufficiently low censoring probabilities relative to the quantile of interest are selected (i.e. the households that did purchase alcohol), defined as $J_0 = \{i\colon pX_i^{'}\hat{\gamma} > 1 - \theta + c$ where θ is the quantile and c is the trimming constant between 0 and 1, set to 0.1 in our case. Following [7], we allow for misspecification of the model by excluding the observations that could theoretically be used but have censoring probabilities in the highest quantiles.

Second, we obtain the initial (consistent but inefficient) estimator $\hat{\beta}_\theta^0$ using standard linear quantile regression, shown in Eq. (4), for the sample J_0. This initial estimator is used to define a new subsample of observations $J_1 = \{x_i^*\hat{\beta}_\theta^0\}$. This sample consists of all observations for which the estimated conditional quantile is above the censoring point. We exclude observations in the lowest quantiles of the distribution of the residuals.

Third, we use standard linear quantile regression, as per Eq. (4), for sample J_1 defined in step two. As shown by [7], this results in a consistent and

efficient estimator of $\hat{\beta}_\theta$. The standard errors of the parameter estimates are obtained with the censored quantile regression bootstrapping procedure described by [8]. For all estimates we use the "cqiv" command available for Stata Version 14 written by [9].

B.2 Counterfactual Analysis

To simulate a counterfactual distribution we use the modelling and inference tools developed by [10]. A complete description of the statistical properties of the

counterfactual estimation methods we implement can be found in [10]. The key element of this approach is the counterfactual unconditional distribution $F_{Y\langle j|k\rangle}$, where Y is the outcome of interest (alcohol purchases), and j and k are the reference (i.e. observed) and counterfactual populations, respectively. $F_{Y\langle j|k\rangle}$ is the distribution of alcohol purchases in population k if it had the same behavioural response (i.e. alcohol purchases) to a change in exogenous characteristics (i.e. per capita household income) as population j. The behavioural response is modelled through the conditional distribution $F_{Y_j|X_j}$, where the index j indicates that this distribution is estimated on the reference population j, and X is a set of covariates. Let F_{X_k} be the unconditional distribution of the covariates in counterfactual population k, then we have

$$F_{Y\langle j|k\rangle}(y) = \int F_{Y_j|X_j}(y|x)dF_{X_k}(x) \tag{10}$$

The counterfactual distribution $F_{Y\langle j|k\rangle}(y)$ is estimated in two steps. First, the conditional distribution $F_{Y_j|X_j}$ is estimated using the censored quantile regression model described earlier, with $\hat{Q}_{Y_j|X_j} = X\beta_j(\theta)$ at various quantiles $\theta \in [0,1]$ using a sample of population j. Next, the conditional distribution is obtained by the relationship

$$\hat{F}_{Y_j|X_j}(y|x) = \int_0^1 1\left\{\hat{Q}_{Y_j|X_j}(\theta|X=x) \leq y\right\} d\theta \tag{11}$$

where $1\{\cdot\}$ is the indicator function and $\hat{Q}_{Y_j|X_j}(\theta|X=x) = x\hat{\beta}_j(\theta)$.

In the second step, the counterfactual unconditional distribution is obtained by using a simple plug-in rule:

$$F_{Y\langle j|k\rangle}(y) = \frac{1}{n_k}\sum_{i\in k}\hat{F}_{Y_j|X_j}(y|x_i) \tag{12}$$

where n is the size of population k and i is an observation in k. This empirical average is the empirical counterpart of the theoretical formula in Eq. (9). Confidence intervals (CIs) can be computed by bootstrap resampling over populations j and k [10]. We further cluster the standard errors at the household level.

Using counterfactual distribution methods we estimate several outcomes of interest. If we partition the vector of covariates X in (inc, Z), then our main counterfactual of interest is the 'after price policy' distribution. The model is estimated for the observed covariates $X_0 = (inc_0, Z_0)$ where inc_0 is the pre-policy per capita income of households and Z is a vector of household characteristics, price indices for alcoholic and non-alcoholic beverages, and quarter dummies that we describe earlier. The counterfactual is obtained for $X_1 = (inc_1, Z_0)$ where inc_1 is the after-policy per capita household income. The before/after unconditional quantile treatment effect (UQTE) can be computed at each quantile θ as

$$\hat{\delta}_p(\theta) = \hat{Q}_{Y\langle X_0,(inc_1,z_0)\rangle}(\theta) - \hat{Q}_{Y\langle X_0,(inc_o,z_0)}(\theta) \tag{13}$$

where $\hat{Q}_{Y\langle j|k\rangle}(\theta) = min\{y\colon F_{Y\langle j,k\rangle}\}(y) \geq \theta$. In practice, the after-policy population is obtained by sampling observations in the current (pre-policy) population, calculating the after-policy tax burden, and setting the observed income inc_0 to the value that it would take when the tax burden from the MUP policy is imposed, as described below. Following this, we can also estimate other policy outcomes of interest, including the predicted change in volume of alcohol (standard drinks) purchased per capita, per quarter (or per day).

For all estimates we use the "counterfactual" command available for Stata Version 14 written by [9]. We have slightly modified this command in order to draw bootstrap samples of households as we work with a panel, and also for clustering the standard errors at the household level.

B.3 Calculating tax burden and adjusted incomes

For Eq. 14, we calculate the value of adjusted per capita household income inc_k at the after policy stage by adapting aspects of the mathematical framework in the Sheffield Alcohol Policy Model (SAPM) version 2.0 (see [11]), including the SAPM method of calculating the expected changes to product prices under a MUP policy and the subsequent change in alcohol purchase costs (i.e. tax burden) for each household. The first step is calculating the current product price (A$) per standard drink (12.67 mL alcohol) as well as the new price under a MUP policy. Using the detailed information in our dataset, we are able to accurately calculate pre-policy values of product prices and household spending using individual details of each product u purchased at each household's separate shopping transactions l, including the alcoholic beverage category k (X 9 categories), container size in litres s, quantity q of containers purchased in the transaction, the alcohol by volume (ABV%) content of each product a_u, and the price paid at the time of purchase p_l. With this information, we calculate the current price paid (A$) per standard drink d for each individual product transaction, denoted $price_{0uld}$ and expressed as:

$$price_{0uld} = \frac{(\frac{p_{ul}}{a_u(q_l(s_{ul})})}{d} \quad (14)$$

To calculate $price_{1uld}$, which is the after-policy price per standard drink that each product will take under the simulated A$2.00 MUP policy, we inflate the value of $price_{0uld}$ up to A$2.00 if it is less than A$2.00, otherwise the price is left unchanged. We derive price indices for $price_{1uld}$ (ex post MUP scenario) for inclusion in our counterfactual analysis model, as described above. The second step is to calculate the additional alcohol purchase costs per capita (i.e. tax burden) for each household at the new reduced quantity, post $2 MUP. We denote exp_{0pli} and exp_{1pli} to be the pre-policy and after-policy purchase costs, respectively, which represent the summed pre-policy $price_{0uld}$ (as defined in Eq.15), and summed after-policy $price_{1uld}$ at all shopping transactions l, for ex post consumption by each individual i aged greater than eleven years within each household j, of alcoholic beverage category k. To calculate the additional annual per capita tax burden T under a MUP policy for each household j we subtract for a specific quantity the pre-policy purchase costs from after-policy purchase costs, expressed as:

$$T_j = (\sum_{k=1}^{K} \frac{exp_{1plik}}{j}) - (\sum_{k=1}^{K} \frac{exp_{0plik}}{j}) \quad (15)$$

The third and final step is to calculate adjusted incomes inc_k for households at the 'after price policy' stage. For this, we simply subtract the annual per capita tax burden for each household (T_j) from each household's pre-policy per capita income inc^*.

B.4 Tables

Table A. Counterfactual analysis: Unconditional quintile treatment effects (UQTE)

		Pointwise	95% CI	
**Quantile**	**UQTE**	**Standard Error**	**Lower**	**Upper**
50	0.0045	0.0421	-0.078	0.0869
51	0.0117	0.0434	-0.0735	0.0968
52	0.0067	0.0442	-0.0799	0.0932
53	0.0158	0.0418	-0.0661	0.0978
54	0.0211	0.0395	-0.0564	0.0985
55	0.0095	0.0389	-0.0668	0.0858
56	0.0162	0.0382	-0.0588	0.0912
57	0.0138	0.0377	-0.0601	0.0877
58	0.0027	0.0347	-0.0654	0.0709
59	0.0015	0.0356	-0.0682	0.0712
60	0.0039	0.0351	-0.065	0.0728
61	0.0209	0.0321	-0.0421	0.0839
62	0.0124	0.0302	-0.0469	0.0716
63	0.0021	0.0293	-0.0554	0.0596
64	0.0087	0.0288	-0.0478	0.0651
65	0.0026	0.0278	-0.0519	0.0571
66	0.0179	0.0279	-0.0367	0.0725
67	0.0029	0.0266	-0.0492	0.055
68	-0.0005	0.0249	-0.0493	0.0483
69	0.0114	0.0246	-0.0369	0.0597
70	0.0052	0.0234	-0.0406	0.051
71	0.003	0.0247	-0.0454	0.0513
72	0.0024	0.0221	-0.041	0.0457
73	0.0035	0.0203	-0.0363	0.0433
74	0.0041	0.0206	-0.0363	0.0444

Continued on next page

Table A – *Continued from previous page*

		Pointwise	95% CI	
**Quantile**	**UQTE**	**Standard Error**	**Lower**	**Upper**
75	0.0048	0.0204	-0.0351	0.0447
76	0.0167	0.0192	-0.0208	0.0543
77	0.0035	0.0188	-0.0333	0.0403
78	0.0157	0.0192	-0.022	0.0533
79	0.0101	0.0184	-0.0258	0.0461
80	0.0112	0.018	-0.0242	0.0465
81	0.0036	0.0183	-0.0322	0.0395
82	0.0336	0.0196	-0.0048	0.0721
83	0.0144	0.0189	-0.0227	0.0515
84	0.0081	0.0195	-0.03	0.0463
85	0.0034	0.0202	-0.0363	0.0431
86	0.0133	0.0219	-0.0296	0.0562
87	0.0132	0.0226	-0.0311	0.0576
88	0.0141	0.0243	-0.0335	0.0618
89	0.0084	0.0257	-0.042	0.0587
90	0.0164	0.0267	-0.036	0.0687
91	0.0122	0.0304	-0.0474	0.0718
92	0.0207	0.0337	-0.0453	0.0866
93	0.0371	0.0376	-0.0367	0.1109
94	0.0107	0.0526	-0.0924	0.1137
95	-0.0117	0.0448	-0.0995	0.0761

Table B. Tax Burden and corresponding change in alcohol purchasing by Quantiles

Additional annual tax burden		Change in standard drinks per day		
			95% CI	
**Quantile**	**Mean (A$)**	**Mean**	**Lower**	**Upper**
50	49.68	0	0.01	-0.01
51	37.64	0	0.02	-0.01
52	65	0	0.02	-0.02
53	14.92	0	0.02	-0.01
54	48.64	0	0.02	-0.01
55	47.48	0	0.02	-0.02
56	47.32	0	0.02	-0.01
57	56.2	0	0.02	-0.02
58	44.4	0	0.02	-0.02
59	85.04	0	0.02	-0.02
60	76.16	0	0.03	-0.02
61	77.2	0.01	0.03	-0.02
62	77.52	0.01	0.03	-0.02
63	70.72	0	0.03	-0.02
64	129.08	0	0.03	-0.02
65	106.32	0	0.03	-0.03
66	115.48	0.01	0.04	-0.02
67	76.52	0	0.03	-0.03
68	114.96	0	0.03	-0.03
69	106.28	0.01	0.04	-0.02
70	115.32	0	0.04	-0.03
71	136.96	0	0.04	-0.03
72	128.12	0	0.03	-0.03
73	121.6	0	0.03	-0.03
74	217.88	0	0.04	-0.03

Continued on next page

Table B – *Continued from previous page*

Additional annual tax burden		Change in standard drinks per day		
			95% CI	
**Quantile**	**Mean (A$)**	**Mean**	**Lower**	**Upper**
75	163.72	0	0.04	-0.03
76	133.76	0.02	0.05	-0.02
77	119.48	0	0.04	-0.03
78	162.2	0.02	0.05	-0.02
79	159.04	0.01	0.05	-0.03
80	168.72	0.01	0.05	-0.03
81	126.52	0	0.05	-0.04
82	166.28	0.04	0.09	-0.01
83	202	0.02	0.07	-0.03
84	266.12	0.01	0.07	-0.04
85	393.28	0.01	0.07	-0.06
86	250.76	0.02	0.1	-0.05
87	335.8	0.02	0.11	-0.06
88	206.32	0.03	0.12	-0.07
89	238.12	0.02	0.13	-0.09
90	552.12	0.04	0.17	-0.09
91	375.4	0.03	0.2	-0.13
92	662.88	0.06	0.27	-0.14
93	511.48	0.13	0.38	-0.13
94	1212.8	0.04	0.45	-0.36
95	1561.72	-0.05	0.34	-0.45

References

1. Zhen C, Finkelstein EA, Nonnemaker JM, Karns SA, Todd JE. Predicting the Effects of Sugar-Sweetened Beverage Taxes on Food and Beverage Demand in a Large Demand System. American Journal of Agricultural Economics. 2014;96(1):1–25.

2. Hausman JA. Valuation of new goods under perfect and imperfect competition. In: The economics of new goods. University of Chicago Press; 1996. p. 207–248.

3. Koenker R. Quantile regression. 38. Cambridge university press; 2005.

4. Buchinsky M. Recent advances in quantile regression models: a practical guideline for empirical research. Journal of human resources. 1998; p. 88–126.

5. Powell JL. Least absolute deviations estimation for the censored regression model. Journal of Econometrics. 1984;25(3):303–325.

6. Powell JL. Censored regression quantiles. Journal of Econometrics. 1986;32(1):143–155.

7. Chernozhukov V, Hong H. Three-step censored quantile regression and extramarital affairs. Journal of the American Statistical Association. 2002;97(459).

8. Bilias Y, Chen S, Ying Z. Simple resampling methods for censored regression quantiles. Journal of Econometrics. 2000;99(2):373–386.

9. Chernozhukov V, Fernández-Val I, Kowalski AE. Censored Quantile Instrumental Variable Estimation via Control Functions. National Bureau of Economic Research Working Paper 16997; 2011.

10. Chernozhukov V, Fernández-Val I, Melly B. Inference on counterfactual distributions. Econometrica. 2013;81(6):2205–2268.

11. Brennan A, Meier P, Purshouse R, Rafia R, Meng Y, Hill-Macmanus D, et al. The Sheffield Alcohol Policy Model–A Mathematical Description. Health economics. 2015;24(10):1368–1388.
